# The *Rhipicephalus appendiculatus* tick vector of *Theileria parva* is absent from cape buffalo (*Syncerus caffer*) populations and associated ecosystems in northern Uganda

**DOI:** 10.1007/s00436-020-06728-x

**Published:** 2020-06-04

**Authors:** I. Obara, N. Githaka, A. Nijhof, J. Krücken, A. Nanteza, D. Odongo, D. Lubembe, P. Atimnedi, D. Mijele, A. Njeri, S. Mwaura, G. Owido, J. Ahmed, P. H. Clausen, R. P. Bishop

**Affiliations:** 1 Institute for Parasitology and Tropical Veterinary Medicine, Freie Universität Berlin, Robert-von-Ostertag-Str. 7-13, 14163 Berlin, Germany; 2 International Livestock Research Institute (ILRI), Nairobi, Kenya; 3 College of Veterinary Medicine, Animal Resources and Biosecurity, Makerere University, Kampala, Uganda; 4 School of Biological Sciences, University of Nairobi, Nairobi, Kenya; 5 Uganda Wildlife Authority, Kampala, Uganda; 6 Kenya Wildlife Service, Nairobi, Kenya; 7 Department of Veterinary Microbiology & Pathology, Washington State University, Pullman, WA USA

**Keywords:** *T. parva*, East Coast fever, *R. appendiculatus*, Uganda, Cape buffalo

## Abstract

*Rhipicephalus appendiculatus* is the major tick vector of *Theileria parva*, an apicomplexan protozoan parasite that causes the most economically important and lethal disease of cattle in East and central Africa. The African cape buffalo (*Syncerus caffer*) is the major wildlife host of *T. parva* from southern Uganda and Kenya to southern Africa. We show herein that *R. appendiculatus* appears to be absent from the two largest national parks in northern Uganda. *Syncerus caffer* is common in both of these national parks, specifically Murchison falls (MFNP) and Kidepo Valley (KVNP). We re-confirmed the previously reported absence of *T. parva* in buffalo sampled in the two northern parks based on RLB data using a nested PCR based on the *T. parva* p104 gene. By contrast, *T. parva*-infected *R. appendiculatus* ticks and parasite-infected buffalo were present in Lake Mburo (LMNP) in South central Uganda. This suggests that the distribution of *R. appendiculatus*, which is predicted to include the higher rainfall regions of northern Uganda, may be limited by additional, as yet unknown factors.

## Introduction

The distribution of ixodid tick species, including *Rhipicephalus appendiculatus*, is believed to be determined primarily by climatic factors, combined with the availability of permissive mammalian hosts on which nymphal and adult ticks can feed to repletion. The distribution of arthropod vectors and wildlife hosts can have serious implications for disease epidemiology and the effectiveness of measures to control livestock pathogens. For example at the wildlife-livestock disease interface, transmission of *Theileria parva* to cattle involves the African cape buffalo (*Syncerus caffer*), the major mammalian wildlife host, that is asymptomatic when infected with *T. parva* by the ixodid tick *R. appendiculatus* (Norval et al. 1992; Young et al. 1973). Buffalo-derived *T. parva* induces a distinct clinical syndrome in cattle when compared to cattle transmissible parasites, characterised by low levels of schizont parasitosis and piroplasm parasitaemia, when transmitted to susceptible animals by *R. appendiculatus* (Norval et al. 1992).

Despite practical issues constraining production and delivery (reviewed by Di Giulio et al. 2009), an infection and treatment (ITM) vaccination procedure is currently the only practical means of providing immunity against *T. parva* in cattle. A large-scale production process for a specific incarnation of ITM known as the Muguga cocktail (Radley et al. 1975) has been refined over the past 20 years (reviewed by Patel et al. 2016). Despite repeated demonstration of efficacy in the laboratory and in the field, ITM delivery has been constrained by regulatory issues, especially in Uganda where only limited vaccination trials have been performed in cross-bred or taurine cattle (Nsubuga-Mutaka 1999).

Recent experimental trials in central Kenya have highlighted the fact that ITM can fail to induce protection in cattle against locally circulating *T. parva* populations in areas where buffalo are also present (Sitt et al. 2015; Bishop et al. 2015). We demonstrate herein that in ecosystems typified by the two National parks in northern Uganda, both the *R. appendiculatus* tick vector and the *T. parva* parasite are not associated with Cape buffalo populations. An earlier study by Oura et al. (2011) using the reverse line blot (RLB) technique detected *T. parva* in buffalo in Lake Mburo national park (LMNP), but not in the blood of buffalo sampled in the Murchison Falls National Park (MFNP) or Kidepo Valley National Park (KVNP). The Oura study, however, did not simultaneously examine the tick populations associated with the buffalo. The data indicates the absence of a buffalo wildlife reservoir of *T. parva* that could infect cattle in the northern Uganda region of East Africa. However, models based on tropical *R. appendiculatus* distribution in sub-Saharan Africa suggest that the habitat in northern Uganda should be favourable for the tick (Perry et al. 1990; Leta et al. 2013). The surprising absence of *R. appendiculatus* in this region suggests a requirement for in-depth investigation of parameters determining the distribution of the tick, with implications for the wider dissemination of *T. parva* and East coast fever in hitherto unaffected regions of sub-Saharan Africa.

## Materials and methods

**Fig. 1:**
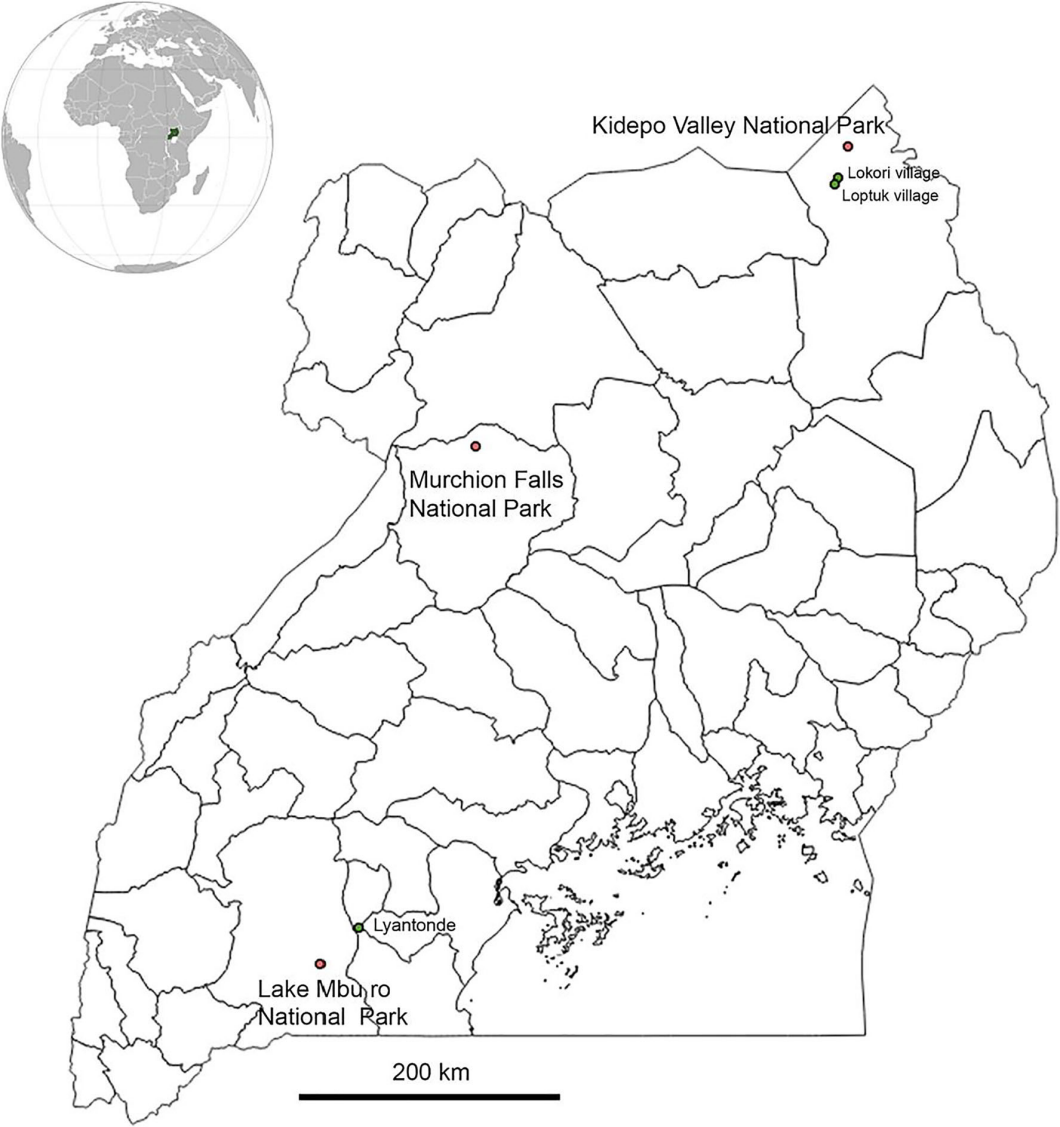
Map showing the sampling sites. The three national parks are indicated with red dots and the cattle sampling sites adjacent to the parks depicted as green dots

Tick sampling involved (i) collection of host-seeking ticks from the vegetation in the early morning and late afternoon by cloth dragging and flagging from LMNP and MFNP in areas where buffalo had recently grazed and (ii) direct collection of engorged, as well as unfed adults from buffalo that were darted in MFNP (*n* = 22) and KVNP (*n* = 33). The location of these parks within Uganda is shown in the map in Fig. 1. The initial tick collections were conducted during August 2016, and repeat sampling in the same locations within the parks was undertaken in January 2017. Tick species identification initially used a morphology-based classification (Walker et al. 2003). Subsequently, the mitochondrial cytochrome oxidase subunit I (COI) gene of ticks was amplified using primers LCO1490 and HC02198 as described (Folmer et al. 1994). Amplicons were purified and bidirectionally sequenced on an ABI sequencer (Macrogen, Korea). Sequence editing and variant calling were performed using the Geneious Prime software (Kearse et al. 2012). For detection of *T. parva*, ticks identified as *R. appendiculatus* were pre-fed on rabbits and their salivary glands were subsequently dissected. Half of a dissected tick salivary gland was stained in Schiff’s reagent following fixation, and infection rates were estimated by counting the number of sporozoites in 70 female and 70 male ticks. Genomic DNA was extracted from the remaining salivary gland sub-sample using a DNeasy Blood & Tissue kit (QIAGEN). For detection of parasite DNA in buffalo, blood samples were collected from the jugular vein of immobilized buffalo and transferred into heparinized tubes for subsequent DNA isolation. Venous blood was also collected from pastoralist Ankole cattle grazing adjacent to buffalo in LMNP at the same time as the buffalo were sampled, and in transhumant Zebu cattle populations from two locations close to KVNP in September 2017. DNA was extracted from heparinized venous blood using the Qiagen kit. In both tick and blood DNA, *T. parva* genomic DNA was detected using nested primers targeting the gene encoding the *T. parva* p104 antigen (Odongo et al. 2010).

## Results and discussion

The distribution of ixodid ticks and *T. parva* infection in buffalo was analysed in MFNP and KVNP in northern Uganda, together with LMNP in South central Uganda. Tick sampling was undertaken in August 2016 (representing the wet season in northern Uganda and a dry season in Southern central Uganda) and again in January 2017 (representing a dry season in both southern and northern Uganda), and the batches of ticks collected at these sampling points were analysed independently. The following tick species were collected directly from buffalo or by dragging grasslands in which buffalo grazed in MFNP: *Rhipicephalus evertsi* (*n* = 116), *Rhipicephalus pravus* (*n* = 7), *Amblyomma variegatum* (*n* = 123), *Amblyomma gemma* (*n* = 63) and species within the genus *Hyalomma* (*n* = 89). The tick species collected from buffalo in KVNP, were *Rhipicephalus evertsi* (*n* = 6), *Rhipicephalus pravus* (*n* = 13), *Amblyomma variegatum* (*n* = 2) and *Amblyomma lepidum* (*n* = 4). However, the major tick vector of *T. parva*, *R. appendiculatus*, was absent from both parks. By contrast, among the ticks collected from LMNP in January 2017, the commonest species was *R. appendiculatus* (*n* = 479), although *Amblyomma cohaerens* (East Africa buffalo tick *n* = 31), *R. evertsi* (*n* = 3), *R. pravus* (24) and *Hyalomma* species (*n* = 13) were also confirmed as being present. It is noteworthy that *R. appendiculatus* and *T. parva* were absent from both MFNP and KVNP during the August wet season in northern Uganda, when tick numbers and parasite transmission intensity would be expected to be at their peak. While detection of both *R. appendiculatus* and *T. parva* in LMNP was during a relatively dry period when tick numbers would not be expected to be at their maximum, the *R. appendiculatus* morphology-based species identification was confirmed by mitochondrial COI sequence-based genotyping (GenBank MN756033-MN756039). Of the six different COI haplotypes identified, five had previously been described in the Great Lakes region (Amzati et al. 2018) and one was novel. A total of 27 of the 140 adult *R. appendiculatus* ticks examined following salivary gland dissection were found to contain *T. parva*-infected acini, and this was confirmed by p104 PCR (data not shown). The identification of other tick species was by morphology only and not confirmed by COI gene analysis.

A nested p104-based PCR was applied to DNA extracted from buffalo blood samples (Odongo et al. 2010). No evidence of *T. parva* infection was detected using the nested PCR assay on buffalo-derived parasite samples from MFNP and KVNP in northern Uganda; however, 45% of the LMNP buffalo were positive. The values of prevalence of *T. parva* in LMNP suggested by the p104 PCR data were lower than those observed in the earlier RLB study in which all animals were infected (Oura et al. 2011). The reasons for this discrepancy are unclear and could be attributable to differences in methodology (use of p104 PCR relative to RLB), sample processing and preservation, seasonality of sampling or a combination of these. It may also be the case that since the Lake Mburo buffalo in this study were sampled in a low tick challenge season, the levels of parasite, which are known to fluctuate in carrier animals, were below the detection threshold. However, in the context of the current study, the key result was that the absence of *T. parva* from buffalo in MFNP and KVNP and presence in LMNP correlated with the distribution of the *R. appendiculatus* tick vector. Among 65 Zebu (*Bos indicus*) cattle sampled from two farms 20–30 km from KVNP, 9% (*n* = 6) were PCR positive for *T. parva*. Among 100 Ankole cattle from farms directly adjacent to LMNP, 56% were positive by p104 PCR. Further north in the Sudan, *T. parva* has been observed to be endemic in cattle, and the presence of the parasite and the vector in this country is thought to be at least partially mediated by extensive anthropogenic movement of livestock (Malak et al. 2012; Marcellino et al. 2015; Salih et al. 2007). In this context, it will be important to examine the *T. parva* infection status of cape buffalo in South Sudan, since ITM vaccination may in future be deployed in this region, and it is possible that buffalo-derived *T. parva* could breakthrough immunity induced in cattle as observed in Kenya. We hypothesise that the infected carrier cattle from the villages close to KVNP that were detected using p104 PCR were imported from the neighbouring Equatoria state in Sudan, rather than infected locally, given the absence of the *R. appendiculatus* vector. There is no evidence that these cattle had ever been associated with buffalo in KVNP.

This distribution of the *R. appendiculatus* vector is surprising since Murchison Falls and Kidepo Valley are both sufficiently humid and well vegetated, and hence climatically and ecologically suitable, for *R. appendiculatus* (Perry et al. 1990; Norval et al. 1992). *Rhipicephalus appendiculatus* ticks can be found up to 8000 ft above sea level in areas with an annual rainfall of over 500 mm (Norval et al. 1992). It is also possible that the current limits of *R. appendiculatus* distribution may have a biotic component, in addition to climate and vegetation, perhaps a result of predation pressure, or competition with a closely related species of tick. It is also important to note that *R. appendiculatus* ticks are under-represented in historical tick collections of wildlife in Uganda (Matthysse and Colbo 1987). The factors determining the distribution of *R. appendiculatus* merit further investigation, particularly since *T. parva* and *R. appendiculatus* are absent from West Africa (Norval et al. 1992) and most of central Africa (Silatsa et al. 2019), although buffalo occur throughout the region.
